# Microbial life in the Lake *Medee*, the largest deep-sea salt-saturated formation

**DOI:** 10.1038/srep03554

**Published:** 2013-12-19

**Authors:** Michail M. Yakimov, Violetta La Cono, Vladlen Z. Slepak, Gina La Spada, Erika Arcadi, Enzo Messina, Mireno Borghini, Luis S. Monticelli, David Rojo, Coral Barbas, Olga V. Golyshina, Manuel Ferrer, Peter N. Golyshin, Laura Giuliano

**Affiliations:** 1 Institute for Coastal Marine Environment, CNR, Spianata S.Raineri 86, 98122 Messina, Italy; 2 Department of Molecular and Cellular Pharmacology, University of Miami Miller School of Medicine, Miami, FL 33136; 3 Institute for Marine Sciences, ISMAR-CNR, Forte S.Teresa, 19136 Pozzuolo di Lerici, La Spezia, Italy; 4 Center for Metabolomics and Bioanalysis, University CEU San Pablo, Boadilla del Monte, 28668 Madrid, Spain; 5 School of Biological Sciences, Bangor University, ECW Bldg Deiniol Rd, Bangor, Gwynedd LL57 2UW, UK; 6 Institute of Catalysis, CSIC, Marie Curie 2, 28049 Madrid, Spain; 7 Mediterranean Science Commission (CIESM), 16 bd de Suisse, MC 98000, Monaco; 8 These authors contributed equally to this work.

## Abstract

Deep-sea hypersaline anoxic lakes (DHALs) of the Eastern Mediterranean represent some of the most hostile environments on our planet. We investigated microbial life in the recently discovered Lake *Medee*, the largest DHAL found to-date. *Medee* has two unique features: a complex geobiochemical stratification and an absence of chemolithoautotrophic Epsilonproteobacteria, which usually play the primary role in dark bicarbonate assimilation in DHALs interfaces. Presumably because of these features, *Medee* is less productive and exhibits reduced diversity of autochthonous prokaryotes in its interior. Indeed, the brine community almost exclusively consists of the members of euryarchaeal MSBL1 and bacterial KB1 candidate divisions. Our experiments utilizing cultivation and [^14^C]-assimilation, showed that these organisms at least partially rely on reductive cleavage of osmoprotectant glycine betaine and are engaged in trophic cooperation. These findings provide novel insights into how prokaryotic communities can adapt to salt-saturated conditions and sustain active metabolism at the thermodynamic edge of life.

Hypersaline environments were widespread during past geological epochs and are still common today. During the Permo-Triassic period the salt-saturated Zechstein Sea covered almost all Northern Europe^1^. The last salinity crisis of Messinian period between 5.6 and 5.3 M years ago was characterized by cyclic desiccation of the Mediterranean Sea that caused deposition of enormous quantities of evaporites^2^. The subsequent dissolution of these ancient evaporite rocks results in the formation of brines that fill seafloor depressions. These formations occur at a depth of more than 3,000 m below the sea level (bsl) and are known as deep-sea hypersaline anoxic lakes (DHALs)^3^,^4^,^5^. The stability of DHALs requires the concurrence of several factors including suitable bottom topology, the presence of permanent evaporites outcroppings and protection from deep-sea currents^5^. DHALs *Discovery*, *L'Atalante* and *Urania* are situated on the accretionary complex of the Mediterranean Ridge (Fig. 1) constituting what is sometimes referred to as the “anoxic lake region”, wherein the existence of yet unknown DHALs was postulated^6^. Indeed, two brine-filled formations, *Thetis* and *Medee*, were recently discovered in this area^7^,^8^,^9^,^10^.

Since the density of salt-saturated brines is high, all DHALs are separated from the overlaying seawater by a thin and very stable pycno/chemo/redoxcline. This isolation prevents both advective and convective exchange of oxygen and other chemical constituents across this boundary^6^. Therefore, the Mediterranean DHAL brines present excellent opportunity to study biogeochemical cycling of the elements and the redox-related diagenetic reactions. The redox boundary at the brine/seawater interface provides energy to various types of chemolitho- and heterotrophic communities. Aerobic oxidations of reduced manganese and iron, sulphide and intermediate sulfur species, diffusing from anaerobic interior to the oxygenated layers are highly exergonic processes capable of supporting the elevated biomass at DHAL interfaces^11^,^12^,^13^,^14^,^15^. Depending on availability of oxygen and other electron acceptors bacterial autotrophic communities belonging to Alpha-, Gamma- and Epsilonproteobacteria fix CO_2_ mainly via the Calvin-Benson-Bassham and the reductive tricarboxylic acid (rTCA) cycles, respectively.

Notwithstanding harsh environmental conditions, Mediterranean DHALs are populated by diverse and very unusual extreme halophilic organisms that belong to all three domains of life^12^,^16^. Many of them were originally discovered in Mediterranean DHALs and named as MSBL (Mediterranean Sea Brine Lakes) candidate divisions^12^,^13^,^15^. DHAL interfaces act as hot spots of deep-sea microbial activity that significantly contribute to *de novo* organic matter production in deep Mediterranean Sea^15^. Metabolically active prokaryotes are sharply stratified across the interface^11^,^13^,^14^,^15^ and likely provide organic carbon and energy that sustain the microbial communities of the underlying salt-saturated brines. Since metagenomic analysis of DHALs is still in its infancy^17^, the metabolic patterns prevailing in the organisms residing in the interior of DHALs remain unknown.

The purpose of this work was to explore the largest known DHAL, Lake *Medee*, which remains uncharacterized with respect to geochemistry and autochthonous microbial diversity. We examined the presence, abundance, ecological significance and trophic interaction of dominant prokaryotic life forms inhabiting the interface and brine. To link *de novo* organic matter production with activity of the resident brine community, we performed [methyl-^14^C]-glycine betaine incorporation experiments followed by enrichment approaches. Our results indicate for the first time that some prokaryotes that belong to MSBL1 and KB1 candidate divisions are involved in trophic cooperation.

## Results

### Geochemical settings in the Lake *Medee*

**Figure 1:**
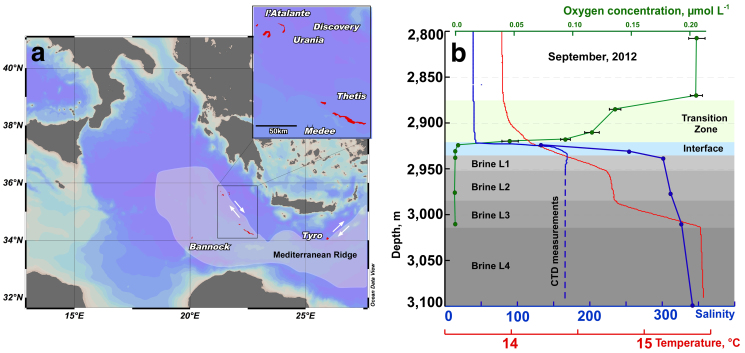
Location of the “anoxic lakes region” on the Eastern Mediterranean Ridge (a) and depth profile of geochemical markers through the Lake *Medee* (b). Arrows indicated strike slip component of motion resulting from strain partitioning. The map was constructed with Ocean Data View software^36^. Salinity (blue line) and oxygen (green line) were measured with on-line sensor until they were out of range (dashed line) where grab samples were used (points, solid line). Data points are mean ± standard error (n = 3).

Lake Medee fills a narrow depression at the Eastern edge of the abrupt cliffs of the small ridge located 70 nautical miles SW of Crete (Fig. 1). This depression is approximately 50 km in length and a rough estimation of the brine surface gave an area of about 110 km^2^ and a volume of nearly 9 km^3^, which places Lake *Medee* among the largest hypersaline formations in the deep-sea environment. At the depth of 2,877–2,880 m, approximately 40 meters above the interface, we detected a slight leap in oxygen concentration, salinity and temperature (Fig. 1). The subsequent analysis of samples collected from this depth confirmed the increase in salinity values from 38.7 to 39.0, whereas oxygen concentration dropped from 212 ± 17 to 133 ± 12 μmol L^−1^. Gradual consumption of oxygen down to 93 ± 9 μmol L^−1^ and an increase in salinity up to 42 were detected within inferior 40 meters (Fig. 1).

The chemo- and pycnocline of Lake *Medee* was found at the depth of 2,920 m. Similarly to other Mediterranean DHALs^9^,^12^,^13^,^14^,^15^, this layer also corresponds to the redoxcline. A complete depletion of oxygen was observed within the upper 70 cm of the interface where the salinity raised from 42 to ≈100 (Fig. 1). Further chemical analyses of the brine samples showed that it consisted of four distinct brine layers (brine L1–L4) with staircase pattern of increasing salinities and temperatures (Table 1). The strongest temperature increase from 14.75°C to 15.46°C was recorded in the 30 m-thick brine L3, at the depths between 2,985 and 3,015 m. Brine L4 is the most saline (salinity, 345) and dense (1.224) (Table 1). The chemical composition shows that Lake *Medee* is a thalassohaline formation with the Na^+^, Cl^−^, Mg^2+^, K^+^ and SO_4_^2−^ being the major ions (Table 1). Hydrogen sulfide was not found in the aerobic part of the interface, but its concentration increased in the anoxic parts reaching the maximum concentration of 1.67 mmol L^−1^ near the seabed. Similarly to other Mediterranean DHALs^13^,^15^,^18^, nitrate was undetectable in the brine, whereas the ammonium concentration drastically increased from less than 0.3 to 2,450 μmol L^−1^ in the anoxic part of the interface and remained virtually constant in the brines.

We studied the geochemical settings and microbial colonization of Lake *Medee* in five consecutive cruises during the period of 2008–2012 at nine different sampling sites (Table S1). Despite the complexity of this hypersaline formation, it was very stable as there were no detectable changes in various parameters (Fig. S1).

### Upper layers of Lake *Medee* rely primarily on autotrophic fixation of bicarbonate

**Figure 2:**
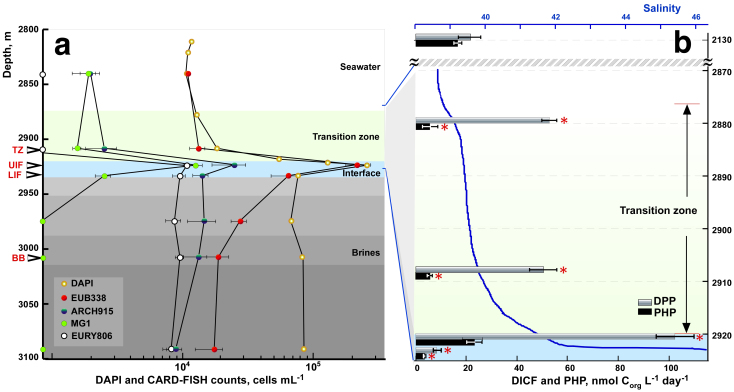
Depth profiles of microbiological abundance and productivity through the Lake *Medee*. Cell counts using DAPI and CARD-FISH (a) and heterotrophic (PHP) and DIC fixation activity (b). Data points are mean ± standard error (n = 3). Significant differences (*p* < 0.05) denoted with asterisks. Layers sampled for molecular analyses indicated by arrows. Abbreviations used: TZ, transition zone; UIF, upper interface; LIF, lower interface; BB, body brine; EUB, Eubacteria; ARCH, Archaea, MG I, Marine Group I of the *Thaumarchaeota*; EURY, *Euryarchaeota*.

Total prokaryote numbers were higher in the *Medee* interface layers (8.6–26.4 × 10^4^ cells mL^−1^) than in the overlying deep seawater and the transition zone (TZ) (1.6–1.9 × 10^4^ cells mL^−1^). The number of cells gradually decreased in the lowermost interface layer and remained relatively constant through all the brines (Fig. 2). Catalyzed reporter deposition fluorescence *in situ* hybridization (CARD-FISH) indicated that while almost all DAPI-stained cells from the interface contained 16S rRNA, the numbers of CARD-positive microorganisms in the brines dropped to less than a half (43.5%) of those visualized by DAPI (Fig. 2, Table S2). Bacteria were predominant in all analyzed samples making up to 90% in less salted layers and up to 62.5% of CARD-positive cells in the *Medee* brine.

We also measured prokaryotic heterotrophic production (PHP) and DIC fixation (DICF), also termed as dark primary production. These measurements were taken at six depth horizons, corresponding to the bathypelagic seawater (2,130 m), upper and lower layers of the *Medee* TZ (2,880 and 2,908 m), upper and middle parts of the *Medee* interface (2,921 and 2,923 m, UIF and LIF, respectively) and the brine L3 (3,010 m) (Fig. 2). Apart from the UIF, the rates of PHP activity in all analysed compartments of *Medee* were significantly lower (*p* < 0.025, ANOVA) than in bathypelagic water masses. No ^3^H-leucine incorporation was detected in salt-saturated brine L3. In contrast to PHP, the 40 m–thick TZ of the Lake *Medee* exhibited a two-fold increase in the DICF activity compared with the bathypelagic water (54 ± 12 vs. 21 ± 9 nmol C_org_ L^−1^ day^−1^, *p* = 0.017, ANOVA). The peak of prokaryote abundance observed at the UIF corresponded to the maximum of DICF activity (102 ± 18 nmol C_org_ L^−1^ day^−1^) (Fig. 2). Two meters below this point bicarbonate fixation rates dropped by an order of magnitude (9 ± 3 nmol C_org_ L^−1^ day^−1^). No DICF activity was detectable in the *Medee* brine L3.

**Figure 3:**
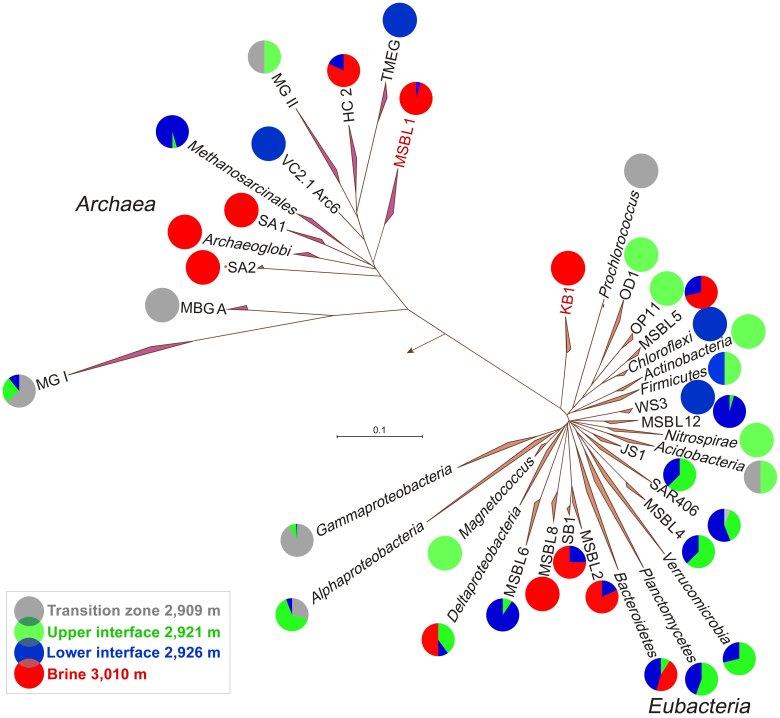
Overview on prokaryotic diversity, stratification and relative abundance of phylogenetic groups recovered from the different compartments of *Medee* Lake. Abbreviations of candidate division used: HC2, Halophilic Cluster 2; KB1, Kebrit Deep Bacteria 1; JS1, Japan Sea 1; MBGA, *Crenarchaea* Marine Benthic Group A; MGI, Marine Group I of the *Thaumarchaeota*; MGII, *Euryarchaea* Marine Group II; MSBLx, Mediterranean Sea Brine Lakes; OD1, OP11-derived 1; OP1 and 11, Obsidian Pool 1 and 11; SA1 and 2, Shaban Deep Archaea 1 and 2; TMEG, Terrestrial Miscellaneous Euryarchaeotal Group; VC2, *Euryarchaea* Hydrothermal Vent; WS3, Wurtsmith aquifer Sequences 3. Stratification and relative abundance of each phylogenetic group found in different layers of the Lake *Medee* is shown as percentage of all cloned sequences related to the indicated group.

To survey the distribution of ribosome-containing *Bacteria* and *Archaea*, we collected total RNA at various depths, which was reverse transcribed, PCR amplified, cloned and a total of 1,001 inserts was sequenced (Table S3). Phylogenetic analysis of the resulting reads revealed a pronounced stratification of prokaryotes thriving in different compartments of the Lake *Medee* (Fig. 3, Fig. S2, Fig. S3). Archaeal communities of both TZ and UIF almost exclusively consisted of members of Marine Group I *Thaumarchaeota* (>96%), whereas the anoxic and more saline LIF and brine L3 were inhabited by different groups of halophilic *Euryarchaeota* with a relatively high abundance of members of *Methanosarcinales* (52% of all LIF clones sequenced). Many bacterial taxa including three candidate divisions MSBL 4, 6 and 12 were flourishing in the LIF (Table S4). Compared to TZ and BB, UIF and LIF possessed the maximum of biodiversity and the highest numbers of missing species (Chao 2 index) (75 and 87, respectively) (Table S5). In the TZ and UIF, the most productive compartments of the Lake *Medee*, the members of SAR11 (*Alphaproteobacteria*) and SUP05 (*Gammaproteobacteria*) clusters constituted up to a quarter of the eubacterial community (Table S4). No riboclones related to *Epsilonproteobacteria* were found in any of the Lake *Medee* compartments.

Our analysis of gene transcripts using RT-PCR supported the notion that microbial populations were stratified phylogenetically and by their involvement in the inorganic carbon assimilation in the TZ and UIF. Transcripts of two sub-forms of RuBisCO, *cbbL*-IAc and -IAq genes, closely related to SUP05 cluster and sulphur-oxidizing facultative autotrophic *Alphaproteobacteria*, were recovered from the oxygenated layers of *Medee* (Fig. S4). Distribution of *aprA* transcripts belonging to *Alphaproteobacteria* and *Gammaproteobacteria* was found very similar to those of 16S rRNA and *cbbL* (Table S4 and Fig. S5). Whereas 100% and 22% of TZ and UIF *aprA* transcripts belonged to sulfur-oxidizing *Gammaproteobacteria*, the *Alphaproteobacteria*-related ones (23%) were found only in UIF layer. Consistent with our phylogenetic analysis (Fig. 3, Fig. S2, Fig. S3), we did not detected any of transcripts of ATP citrate lyase (*acl*A), the key enzyme of the rTCA cycle operated in chemolithotrophic *Epsilonproteobacteria*.

### Contribution of osmoprotectant glycine betaine to microbial life and the trophic network in *Medee* interior

Prokaryotic community thriving in the *Medee* brine was characterized by the overwhelming dominance of archaeal MSBL1 and bacterial KB1 candidate divisions (87 and 72% of all archaeal and bacterial clones, respectively). The prevalence of these yet uncultured organisms in the entire prokaryotic brine population was confirmed by specific CARD-FISH counting. Almost 50% of Eury806-positive cells of *Euryarchaea* (4.6 ± 1.1 from 9.5 ± 2.0 × 10^3^ cells mL^−1^) hybridized with the specific MSBL1 probe (Tables S3, S6 and Fig. S6). While the metabolic preferences of KB1 organisms remain enigmatic, there are some recent assumptions that MSBL1 could be a group of putative methanogens^15^,^19^,^20^. Indeed, we found that methanogenesis occurs within the entire *Medee*'s interior, with some activity present even at the salinity as high as 345 (Table 1). Recovery of methyl coenzyme M reductase (*mcrA*) transcripts confirmed that methanogenic pathway is active in the *Medee* brine. Like *mcrA* transcripts recovered from the brine of another DHAL, Lake *Thetis*, all *Medee* *mcrA* sequences formed a tight cluster distantly affiliated with those from *Methanohalophilus* (Fig. S7). Given the *Methanohalophilus*-related 16S rRNA gene sequences were not found in the *Medee* brine, the methanogenic form of *mcrA* is proposed to be of the MSBL1 origin.

Since the type of substrates fuelling methane production in DHALs is unknown, we monitored the presence of free methylated amines, volatile fatty and amino acids in the *Medee* brines. Neither mono-, nor di- and trimethylamines (TMA) were detected in the brine or sediments of *Medee*. Volatile fatty acids were represented only by acetate. Interestingly, acetate accumulation exhibited bottom-ward trend, increasing from 132 μmol L^−1^ in upper brine to more than 500 μmol L^−1^ in deepest brines. We did not detect any amino acids except glycine betaine (GB) which was abundant both in the interface and the upper brine (81 and 170 nmol L^−1^, respectively), but decreased with depth and was not detectable in the sediments.

**Figure 4:**
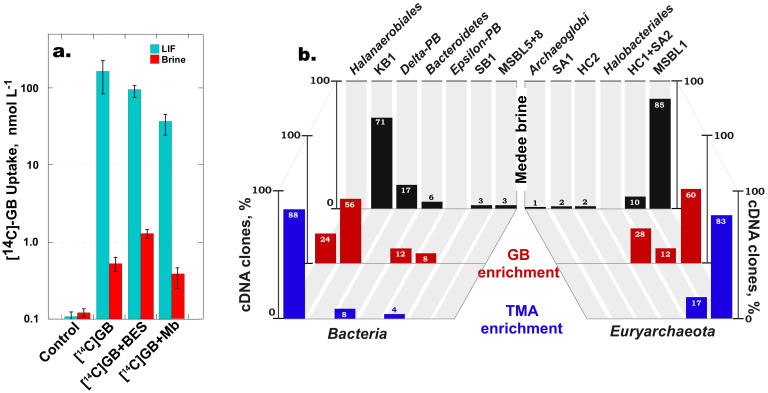
Biochemical and phylogenetic analyses of Lake *Medee* natural community and GB-and TMA-supplemented enrichment. (a). [^14^C-methyl]-GB uptake and (b). Relative abundance of phylogenetic groups obtained from *Medee* brine and GB- and TMA-supplemented enrichments. Measurements are mean ± standard error (n = 3).

To test the hypothesis that GB can support microbial activity in the salt-saturated interior of *Medee*, we applied radiotracer and enrichment analyses (Text S1). In the first approach, we added [^14^C-methyl]-GB to the samples collected from LIF and brine. After one month of incubation, almost 40% of the radioactivity incorporated into the LIF microbial biomass (Fig. 4). The uptake of [^14^C-methyl]-GB was also detected in the brine sample albeit to a lesser extent, evidently because the extreme salinity of this fraction inhibits metabolic activity. Addition of inhibitors of methanogenesis (100 mmol L^−1^ bromoethanesulfonate) and sulphate reduction (20 mmol L^−1^ sodium molybdate) did not significantly affect the GB uptake in either sample (Fig. 4).

In the enrichment experiments, supplementation of brine samples with either 1 mmol L^−1^ GB or 1 mmol L^−1^ TMA under the *in situ* salinity did not cause the biomass increase. However, when we diluted the samples to the values corresponding to the salinity of LIF, the cell density in GB-supplemented enrichment increased 40-fold. Analysis of volatile metabolites revealed the appearance of almost 80 μmol L^−1^ TMA, whereas this tertiary amine was not detected in the original brine sample (Table 2). Methane and acetate concentrations also significantly increased compared to the *in situ* brine values (99 and 693 μmol L^−1^, respectively). Enrichment cultures supplemented with TMA exhibited cell density increase up to 7.7 × 10^6^ cells mL^−1^. Noteworthy, there was a moderate increase in methane production and a striking increase in acetate (41 and 1,291 μmol L^−1^), indicating that more than 75% of the added TMA was converted to this end product (Table 2).

## Discussion

The formation of brine lakes from dissolving evaporites requires a suitable bottom topology and protection from currents^5^,^21^. The *Medee* Basin is flanked from the South by a steep 500–700 m wall, which presumably contains the evaporite rocks. As these evaporites dissolve, the dense brines flow down onto the basin floor where they are shielded from current-induced mixing and dilution. As this process continues, the brines progressively increase their density and became more and more protected and stable^5^,^18^,^21^. The lowermost brine layer L4 of *Medee* is, indeed, the most saline and dense, whereas the surface of lake is covered by a “transition” zone which is slightly more salted and dense than seawater. The presence of such hydrological formation was not shown before in any Mediterranean DHAL and thus seems to be a unique feature of this lake^7^,^8^.

Another unique feature of the Lake *Medee* ecosystem is its low activity in uptake of [^14^C]-bicarbonate (Fig. 2). The ion composition of *Medee* brines (Table 1) is very similar to that of the much more productive thalassohaline Mediterranean DHALs, *L'Atalante* and *Thetis*^9^,^11^. Thus, it is unlikely that ion composition is the main contributor to the low DIC fixation activity in the *Medee* interface. We hypothesized that the differences between *Medee* and other DHALs were due to the presence of functionally distinct microbial populations in *Medee*. We tested this hypothesis by analysing ribosomal and messenger RNA isolated from organisms found in different *Medee* layers. While this approach benefits from the increased stability of allochthonous DNA under DHAL conditions^14^, processing of the samples inevitably results in biases in recovery of mRNA transcripts. Cast recovery, on-board filtration and fixation processes take up to 5 h and therefore influence quantitative aspects of the gene expression analysis. Because of this limitation we only performed qualitative interpretation of targeted transcripts recovery.

Consistent with the 16S rRNA analysis, we detected neither transcripts nor the genes of ATP citrate lyase, the key enzyme of the reductive TCA cycle operating in chemolithotrophic *Epsilonproteobacteria*. The absence of this important group of chemoautotrophic organisms was only documented previously for Lake *Bannock*^13^. Like *Medee*, *Bannock* has a diffuse, 3.0–5.0 m thick interface, a feature distinguishing these two lakes from all other Mediterranean DHALs possessing much thinner (<1.5 m) interfaces. As it has been recently shown^22^,^23^, deep-sea chemoautotrophic *Epsilonproteobacteria* are adapted to highly dynamic and sharp environmental gradients where they could out-compete the less-productive sulphur-oxidizing chemoautotrophic *Gammaproteobacteria*. Thus, the diffuse nature of *Medee* and *Bannock* interfaces can explain the domination of deep-sea *Gammaproteobacteria* over *Epsilonproteobacteria*. We cannot rule out contribution of other factors, but a similar trend in reduction of autotrophic activity was observed in the Black Sea upon the seasonal water column turbulence, which caused a drastic widening of redoxcline^24^.

Among the main factors that determine whether the microorganisms can live at high salinity are the availablity of energy generated during their dissimilatory metabolism and the mode of maintaining the osmotically balanced and functional cytoplasm^19^. Osmotic adaptation could be achieved either through the biosynthesis of organic compatible solutes (osmoprotectants) or accumulation of monovalent ions in cytoplasm (“salt out” and “salt in” strategies)^19^,^25^. The first type of haloadaptation requires high energetic costs and limits the number of prokaryotes that can thrive in salt-saturated environments. These constraints are especially restricting in the dark and anoxic ecosystems where photosynthesis, aerobic respiration and other highly exergonic processes cannot be operative. Therefore, only few anaerobes capable of functional activity at salt saturation are known^1^,^19^,^25^.

As it was recently shown, some of the anaerobic extreme halophiles are syntrophically linked, thus essentially constituting a single functional entity within microbial communities occupying hostile niches^26^,^27^. Established trophic network is based on the reductive degradation of glycine betaine, a common osmoprotectant ubiquitously produced by a variety of moderate “salt out” halophiles residing in the moderately saline layers. Two organisms, an extremely halophilic homoacetogen *Acetohalobium arabaticum* and methylotrophic methanogen *Methanohalobium evestigatum* isolated from hypersaline anoxic sediments of Arabat spit (East Crimea, Ukraine) were involved in GB-based trophic chain^28^. Like other members of *Halanaerobiales*^29^, *A. arabaticum* degrades GB using hydrogen as an electron donor in reductive branch of the Stickland reaction and produces acetate and trimethylamine (TMA), which is further used by a methanogenic member of consortium. In co-culture containing 15% of NaCl, the hydrogen consumption by *A. arabaticum* pushes *M. evestigatum* to divert up to 58% of its reducing power away from methane production into reactions for CO_2_ and H_2_ generation^28^.

Consistently with the low gross DICF, the methane production rates of the *Medee* brine were the lowest among all studied thalassohaline DHALs^12^,^13^,^14^,^15^. However, some activity was detectable even in deepest brine under salt saturation, indicating that methanogenesis may be one of the few operating processes that contribute to the carbon cycling in the Lake *Medee* interior. The recovery of methyl coenzyme M reductase (*mcrA*) gene transcripts confirmed that methanogenic pathway is active in Lake *Medee* brine. We propose that the evolution of methane in *Medee* brines might be the result of reductive degradation of GB and subsequent methanogenic fermentation of the resulting TMA.

This hypothesis is supported by the following observations. First, GB was abundant both in the interface and the upper brine, but decreased with depth and was not detectable in *Medee* sediments, presumably because of its complete consumption. Second, acetate exhibited the opposite trend, accumulating in the deepest brines of the Lake, providing another line of evidence for the proposed acetogenic GB fermentation. Third, it is well documented that neither dimethyl- nor monomethylamines can be used by any of known methylotrophic acetogens^19^,^28^. Since neither TMA, nor dimethyl- or monomethylamines were detected in the brine and sediments of *Medee*, this fact could suggest their complete consumption via methanogenic fermentation.

To obtain direct experimental evidence that GB could support the microbial activity in *Medee* brine, we applied radiotracer and enrichment approaches (Fig. 4). Our experiments with [^14^C-methyl]-GB supported the idea that trophic network in the salt-saturated brines of DHALs may rely on decomposition of this ubiquitous osmoprotectant. Addition of sodium molybdate, an inhibitor of sulphate reduciton^26^ did not affected GB uptake, indicating that this process involved organisms other than sulphate-respiring ones. Bromoethanesulfonic acid (BES) did not inhibit GB uptake at LIF salinities, while slightly stimulating this process in the brine. This finding is in accordance with the observation that at applied concentrations BES mainly inhibited the reductive, CH_4_–producing branch of Wood-Ljungdahl pathway and did not influence the activity of oxidative branch leading to CO_2_ and H_2_ evolution^30^,^31^. Hence, addition of BES did not influence H_2_ production needed for the reductive degradation of GB by an acetogenic fermenter.

Additional evidence for GB contribution to the trophic network of *Medee* brine microbial community was obtained by analysis of brine enrichments (Fig. 4). Supplementation with GB not only significantly increased cell density, but the grown microbial population was similar to that observed in the natural community of the Lake *Medee* brine. The emergence and prevalence of KB1 group is likely to be specifically linked to the availability of GB, because no KB1-related organisms were detected in the TMA-enrichment (Fig. 4).

While the archaeal patterns resembled those of the original brine, supplementation with TMA shifted bacterial population almost exclusively to the members of order *Halanaerobiales*, which may be attributed to a cultivation-biased succession of prokaryotic community^13^,^32^. To date, this group of obligate anaerobic fermenters is considered to be the most halophilic among all cultivable bacterial taxa. This feature is mainly due to their “salt in” osmoadaptation strategy^19^. Similarly to halophilic archaea, these organisms minimize the energy costs by accumulating monovalent ions (mainly potassium and chloride) rather than relying upon the synthesis of organic osmoprotectants (“salt out” osmoadaptation strategy). Noteworthy, some of the cultivated *Halanaerobiales* gain energy via methylotrophic acetogenic modification of reductive acetyl-CoA pathway^1^,^19^,^28^,^29^. *Halanaerobiales*-related organisms have never been detected in DHAL brines by conventional 16S rRNA gene-based phylogenetic analyses^11^,^12^,^13^,^14^,^15^ or metagenomic approaches^17^. Therefore, it is possible that the KB1 group may possess *Halanaerobiales* metabolic patterns.

**Figure 5:**
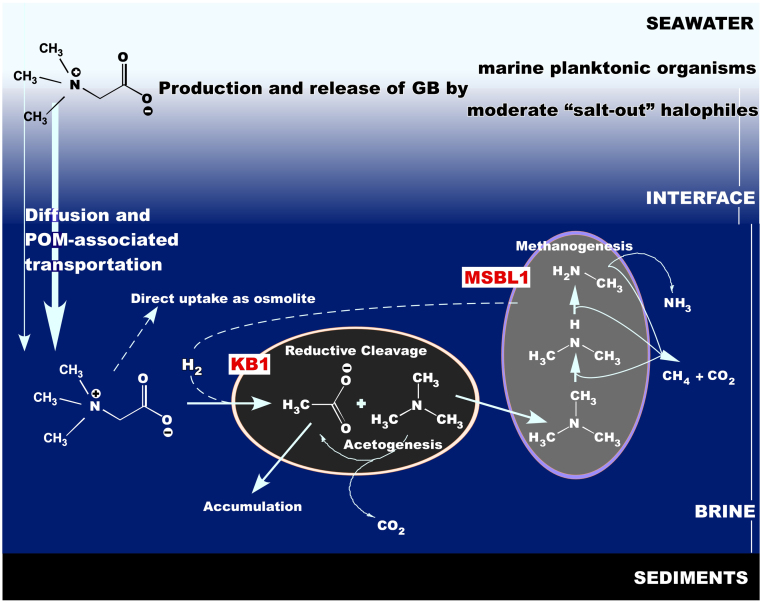
Proposed GB-based C_1_-trophic relations established between the members of KB1 and MSBL1 candidate divisions in salt-saturated brines of the Lake Medee and other Mediterranean DHALs.

In summary, our data allowed us to propose a model explaining how the microbial community in the *Medee* brines operates under conditions incompatible with life of common marine and moderate halophilic microorganisms (Fig. 5). In this stratified ecosystem, the moderately saline compartment overlaying the lake is dense in microbial biomass and is chemolithotrophically active. It supplies life in lower layers with small organic molecules such as GB. The salt-saturated *Medee* brine is dominated by MSBL1 and KB1, yet-uncultured deep-branching candidate divisions. Their activity is most likely carried out by the KB1 members and is based on reductive cleavage of GB, resulting in formation of TMA and acetate. TMA in turn supports the extremely halophilic methylotrophic H_2_-leaking methanogens belonging to the MSBL1 candidate division. We hypothesize that members MSBL1 and KB1, which are ubiquitously found in hypersaline anoxic environments, are the primary beneficiaries of organic matter produced in the overlaying strata.

## Methods

### Sampling in Lake *Medee*

Depth-stratified brine samples were collected on board the RV *Urania* during five consecutive oceanographic cruises during 2008–2012 (Table S1). Sampling was performed with 12 L Niskin bottles, housed on a rosette provided by SBE-911 plus CTD sensors (Sea-Bird Electronics, Bellevue, WA, USA). The interface was captured and fractionated as described elsewhere^13^,^15^. Determination of oxygen concentration and redox potential at chosen depths was carried out following previously established protocols^9^,^13^,^15^.

### Geochemical analyses and salinity-related measurements

Samples for determining major ion concentrations were collected in 1000 mL dark polyethylene (DPE) vials and stored at room temperature. Alternatively, 100 mL of the samples were acidified with 100 mmol L^−1^ of HNO_3_, diluted to salinity 3.5 and stored in DPE vials at room temperature prior to chemical analyses as previously described^9^,^12^,^13^,^14^,^15^. Major elements in brines were analysed by ICP-AES at Syndial S.p.A (Priolo Gargallo, Italy). Hydrogen sulphide, volatile fatty acids and nutrient concentrations were determined as previously described^9^,^12^,^13^,^14^,^15^.

### Activity measurements

Methane production rates were determined as previously described^12^,^13^,^14^,^15^. The DICF was estimated by the incorporation of [^14^C]-bicarbonate (10 μCi mL^−1^, Amersham), according to the methods for aerobic and anoxic measurements respectively^11^,^33^,^34^ (Text S2). Prokaryotic heterotrophic carbon biomass production was estimated using [^3^H]-leucine uptake by the micro-method. For anoxic analysis, an oxygen-free [^3^H]-leucine solution was used and the incubation was carried out in 2 mL plastic syringes (Discardit II, BD). A one-way ANOVA test was applied to determine statistical significance of measured PHP and DICF activities. The relative importance of each value was investigated by Holm-Sidak multiple comparisons method. Calculations were carried out using SigmaStat: Statistical Analysis Software for Windows, ver. 3.1 (Systat Software). Differences were considered significant at *p* < 0.05. Details on *in situ* determination of GB concentration and an uptake of [methyl-^14^C]-glycine betaine are given in Text S1 and S3. Acetate measurement was performed with an electrophoretic buffer that contained phosphoric acid (0.2 mol L^−1^, pH 6): methanol (9:1), as in^35^. GB, methylamines and free amino acids concentrations were determined by using LC-ESI-QTOF-MS as it is described in the supplementary information. The resulting data were cleaned of background noise and unrelated ions by the Molecular Feature Extraction tool in the Mass Hunter Qualitative Analysis software (B.05.00, Agilent). The total TIC (Total Ion Chromatogram) was examined for GB and TMA MS signatures. GB concentration was determined using GB standard (Sigma-Aldrich, Taufkirchen, Germany) with a concentration ranging from 1 to 10 μmol L^−1^.

### CARD-FISH and DNA/RNA-based analyses

For CARD-FISH analysis, samples were fixed with formaldehyde (2% v/v final concentration, for 1 h at room temperature. Cells were permeabilized with lysozyme (10 mg mL^−1^, 1 h) and achromopeptidase (5 mg mL^−1^, 30 min) at 37°C. Intracellular peroxidase were inhibited by treatment with HCl (10 mmol L^−1^) at room temperature for 20 min. We used the following horseradish peroxidase labeled probes: CREN537, EURY806, and EUBI-III. The FISH probes KB1, Delta-DHAL, Halo1192 and MSBL411 were designed in this study (Table S7). The nonspecific probe NON338 did not detect any cells. The filters sections were counter-stained with DAPI (2 μg mL^−1^) in a 4:1 ratio of Citifluor (Citifluor Ltd, Leicester, UK) and Vectashield (Linaris GmbH, Wertheim-Bettingen, Germany). At least 200 DAPI cells, in a minimum of 10 fields, were counted in the AXIOPLAN 2 Imaging microscope (Zeiss). To filter 5–10 L samples we used sterile Sterivex cartridges (0.2 μm pore size, Millipore) which were further stored at −20°C in 400 μL of sterile TE buffer (containing 5 μg mL^−1^ of lysozyme) and 1,600 μL of QRL1 buffer (Qiagen, Milan, Italy). Cartridges were cracked, opened and the filters and lysis buffer were transferred into 50 mL centrifuge tubes.

The simultaneous extraction of nucleic acids was carried our using RNA/DNA mini kit (Qiagen, Milan, Italy) as previously described^9^,^11^. The quality of DNA samples was examined by agarose gel electrophoresis and concentration was determined using NanoDrop ND-1000 Spectrophotometer (Wilmington, DE, USA). The amount of total RNA was also estimated by NanoDrop ND-1000 and further treated with Turbo DNA-free kit (Ambion, Austin, TX, USA). cDNA synthesis was performed from 80 ng of total RNA using SuperScript II Reverse Transcriptase (Invitrogen, Carlsbad, CA, USA) and hexarandom primers according to the manufacturer instructions. The reaction was carried out in a MasterCycler 5331 Gradient (Eppendorf, Hamburg, Germany). Bacterial and archaeal 16S rRNA, *cbbL*, *aclA*, *aprA*, *dsrAB* and *mcrA* cDNA were amplified from obtained cDNA by PCR using primers listed in the Supplementary Table S6. The conditions for RT-PCR and cloning were conducted as described elsewhere^9^,^11^. Possible DNA contamination of RNA templates was routinely monitored by PCR amplification of RNA aliquots that were not reverse transcribed. No contaminating DNA was detected in any of these reactions. Sequence analysis and phylogenetic reconstruction were performed as described in Supplementary Text S4 and S5.

### GB- and TMA-supplemented enrichment

An attempt to enrich the GB- and TMA-degrading microorganisms was performed with the *Medee* brine samples (salinity, 320) collected at depth of 3,010 m in September 2012. Similarly to [methyl-^14^C]-GB experiments, the enrichment cultures were established into serum vials (120 mL) carefully flushed with nitrogen to remove any oxygen. The collected *Medee* brine was transferred into the serum vials prefilled with various volumes of the artificial brine to attain a gradual decrease in final salinity: 300; 278; 254; 230 and 212. Prior to adding the brine samples, 20–90 mL of sterile artificial anoxic brine medium were dispensed to each vial. The medium has the following composition: NaCl 200 g L^−1^; KH_2_PO_4_ 0.33 g L^−1^; yeast extract 0.05 g L^−1^; Na_2_S 2.5 g L^−1^; GB or TMA 1 mmol L^−1^; 10 mL L^−1^ trace elements solution (DSMZ medium 320); and 10 mL L^−1^ vitamin solution (DSMZ medium 141); pH values were adjusted to 6.7 corresponding to *in situ* values of the brine. The amount of the Medee brine varied between 10 and 80 mL to result in the final volume of 100 mL for every enrichment; every enrichment was performed in triplicate. Samples were incubated at *in situ* temperature (16°C) in the dark and checked microscopically weekly. After 30 day incubation, 10 mL and 1 mL of the most saline positive enrichments were taken for DNA isolation and CARD-FISH analysis, respectively. For control we used brine samples diluted to same salinity but without any addition of both GB and TMA. After one month of exposition, these controls exhibited a three-fold decline of initial cell density and amounts of rRNA isolated from 100 mL were insufficient to perform RT-PCR.

### Data deposition

The nucleotide sequences produced in the present study have been deposited in the DDBJ/EMBL/GenBank databases under accession numbers: JX446209 to JX446296 for the archaeal 16S rRNA gene sequences, JX446015 to JX446208 for the bacterial 16S rRNA gene sequences, JX446014 for the archaeal *mcrA* gene sequence, JX445989 to JX446007 for the bacterial *aprA* gene sequences, JX446008 to JX446013 for the bacterial *dsrA* gene sequences and KC424645 to KC424651 for the 16S rRNA gene sequences recovered in GB enrichment.

## Author Contributions

M.M.Y., V.L.C., V.Z.S., P.N.G. and L.G. planned the experiments and carried them out; M.B. and L.S.M. completed the isotopic studies; G.L.S., O.V.G. and E.A. completed the molecular biological studies; M.F., D.R. and C.B. conceived and performed the analytical experiments to identify glycine betaine in brine and sediments; E.M. and M.F. performed the bioinformatics studies; M.M.Y., P.N.G. and V.Z.S. wrote the paper and all authors provided editorial comments. M.M.Y., V.L.C., V.Z.S., M.F. and P.N.G. contributed equally to the work.

## Supplementary Material

Supplementary Information

## Figures and Tables

**Table 1:** Average chemical composition of the *Medee* Lake brines. All concentrations in mmol kg^−1^ unless otherwise stated. Reported geochemical values are mean ± 5% (n = 6) obtained during 2010–2012 years unless otherwise stated. Abbreviations used: GB, glycine betaine; MPR, methane production rates; n.d., not determined

Parameters	Brine L1 2,940 m	Brine L2 2,975 m	Brine L3 3,010 m	Brine L4 3,102 m
Density, kg dm^−3^	1.19	1.21	1.22	1.22
Temperature, °C	14.45	14.73	15.32	15.44
Salinity	304	314	325	345
Na^+^	4,022	4,110	4,165	4,178
Cl^−^	4,684	4,833	4,830	5,259
Mg^2+^	603	630	773	788
K^+^	331	363	462	471
Ca^2+^	2.4	2.6	3.0	2.8
SO_4_^2−^	140.4	146	166.9	201
HS^−^	0.67	0.93	0.97	1.64
Br^−^	49.0	53.3	62.6	65.3
H_3_BO_3_	1.9	2.0	2.2	2.3
NH_4_^+^	2.31	2.27	2.45	2.35
Li^+^ μmol L^−1^	149	160	166	163
CH_4_ μmol L^−1^	18.0 ± 3.1	70.3 ± 2.3	24.1 ± 3.3	13.9 ± 1.4
Acetate μmol L^−1^	132 ± 21	539 ± 42	508 ± 37	n.d.
GB nmol L^−1^	170 ± 9	n.d.	44 ± 7	0*
MPR, μmol L^−1^day^−1^	2.1 ± 0.2	3.1 ± 0.4	1.5 ± 0.6	0.5 ± 0.4

*The values correspond to the glycine betaine concentration found in the sediments collected at the depth of 3,105 m.

**Table 2:** Metabolites analysis and CARD-FISH counting of *Medee* brine grown enrichments supplemented with 1 mmol L^−1^ of GB and TMA. Values are mean ± standard error (n = 3)

Parameters						
	Methane	Acetate	TMA	Free AA	*Archaea*	*Eubacteria*
	μmol L^−1^	μmol L^−1^	μmol L^−1^	μmol L^−1^	×10^4^ cells mL^−1^	×10^4^ cells mL^−1^
Brine, 3,010 m	24 ± 3	508 ± 23	0	0	1.2 ± 0.4	2.0 ± 0.8
GB-Enrichment	99 ± 4	693 ± 13	79 ± 3	n.d.*	47.3 ± 8.3	382 ± 37
TMA-Enrichment	41 ± 3	1,291 ± 14	n.d.	n.d.	53.9 ± 11.3	717 ± 112

*n.d., not determined.
