# Supplementary material for: Microbial life in the Lake Medee, the largest deep-sea salt-saturated formation
Source: Sci Rep. 2013 Dec 19;3:3554. doi: 10.1038/srep03554 (PMC3867751; doi:10.1038/srep03554)
Supplement: Supplementary Information [file srep03554-s1.doc]

**Supplementary Information to manuscript**

**Microbial life in the Lake *Medee*, the largest deep-sea salt-saturated formation*.***

Michail M. Yakimov, Violetta La Cono, Vladlen Z. Slepak, Gina La Spada, Erika Arcadi, Enzo Messina, Mireno Borghini, Luis S. Monticelli, David Rojo, Coral Barbas, Olga V. Golyshina, Manuel Ferrer, Peter N. Golyshin, and Laura Giuliano.

**Supplementary Tables**

**Supplementary Table 1.** The list of the cruises and sampling sites studied during 2008-2012 at the Lake *Medee*.

| **Cruise** | **Date** | **Coordinates** | **Site name** |
| --- | --- | --- | --- |
|  |  |  |  |
| Middle | 02 October 2008 | 34°24.015N 22°26.981E | M31 |
|  |  | 34°19.718N 22°33.594E | M31_BxC |
|  |  |  |  |
| Middle&Mamba | 13 September 2009 | 34°19.780N 22°31.345E | LS4_Medee |
|  |  | 34°19.431N 22°36.623E | LS5_Medee |
| Mamba2010 | 17 June 2010 | 34°19.794N 22°31.373E | SS5_Medee |
| Mamba_C2011 | 16 September 2011 | 34°28.600N 22°15.823E | SC6a_Icast |
|  |  | 34°28.587N 22°15.849E | SC6a _IIcast |
|  |  |  |  |
| Microdeep2012 | 24 September 2012 | 34°19.590N 22°33.644E | MD4_Medea |
|  |  | 34°21.131N 22°27.937E | MD4_Medea |

**Supplementary Table S2. Abundance of general and specific phylogenetic groups of *Bacteria* and *Archaea* kingdoms in the water column and in the Lake *Medee.***

Cells were collected from the indicated depths and hybridized with the specific CARD-FISH probes. For each group, the top value shows the total number of cells (x 10^4^ mL^-1^) of sample, whereas value below shows the percentage of total cells in the given samples.

| **DEPTH**  **(m)** | **EUB338**  **(%)** | **ARCH915**  **(%)** | **CREN537**  **(%)** | **EURY806**  **(%)** | **HALO1192**  **(%)** | **KB1** |
| --- | --- | --- | --- | --- | --- | --- |
|  |  |  |  |  |  |  |
| WC  (2,840) | **1.2 ± 0.18**  **(77%)** | **0.2 ± 0.08**  **(18%)** | **0.2 ± 0.04**  **(15%)** | **0.05 ± 0.01 (2%)** | **0** | **0** |
|  |  |  |  |  |  |  |
| TZ  (2,909) | **1.32 ± 0.23**  **(68%)** | **0.24 ± 0.07**  **(12.5%)** | **0.16 ± 0.02**  **(3%)** | **0.08 ± 0.02 (6%)** | **0** | **0** |
|  |  |  |  |  |  |  |
| UIF  (2,921) | **21.1 ± 4.72**  **(80%)** | **2.5±0.86**  **(10%)** | **1.27±0.25**  **(5%)** | **1.13±0.17 (5%)** | **0** | **0** |
|  |  |  |  |  |  |  |
| LIF  (2,926) | **6.6± 0.8**  **(77%)** | **1.4±0.2**  **(19%)** | **0.25±0.08**  **(3%)** | **0.86±0.02 (12%)** | **0.29±0.06 (4%)** | **0** |
|  |  |  |  |  |  |  |
| BB  (3,010) | **2.0±0.78 (24%)** | **1.2±0.38 (14%)** | **0.09±0.01**  **(1%)** | **0.95 ± 0.20 (12%)** | **0.46±0.11 (6%)** | **0.68±0.20 (9%)** |

The contribution of each group (expressed as %) to total cell abundance revealed by DAPI staining is given in brackets

**Supplementary Table S3.** General information about the *Medee* clone libraries created and number of clones analyzed through the study.

| **Organisms** | **Gene** | **Molecule** | **TZ** | **UIF** | **MIF** | **BB** | **Total** |
| --- | --- | --- | --- | --- | --- | --- | --- |
| ***Bacteria*** | *16S rRNA* | cDNA | 112 | 78 | 102 | 110 | **402** |
|  | *cbbL* | cDNA | 24 | 24 | 24 | 0 | **72** |
|  | *aprA* | cDNA | 24 | 24 | 24 | 24 | **96** |
|  | *dsrAB* | cDNA | 0 | 24 | 24 | 0 | **48** |
| ***Archaea*** | *16S rRNA* | cDNA | 127 | 51 | 76 | 105 | **359** |
|  | *mcrA* | cDNA | 0 | 0 | 0 | 24 | **24** |
| **Total** |  |  | **287** | **201** | **250** | **263** | **1001** |

**Supplementary Table S4.** Phylogenetic affiliation and distribution of the 16S rRNA clones obtained in eight clone libraries of the Lake *Medee*. Abbreviations used: TZ, transition zone; UIF, upper interface; LIF, lower interface; BB, Body brine.

| **Bacteria** | **TZ** | **UIF** | **LIF** | **BB** |
| --- | --- | --- | --- | --- |
| Acidobacteria | **1** | **1** | **0** | **0** |
| Actinobacteria | **0** | **3** | **0** | **0** |
| Alphaproteobacteria | **5** | **11** | **1** | **0** |
| Bacteroidetes | **0** | **1** | **5** | **5** |
| Chloroflexi | **0** | **0** | **1** | **0** |
| Deltaproteobacteria | **0** | **16** | **4** | **20** |
| Firmicutes | **0** | **1** | **1** | **0** |
| Gammaproteobacteria | **104** | **8** | **1** | **0** |
| JS1 | **0** | **2** | **4** | **0** |
| KB1 | **0** | **0** | **0** | **79** |
| Magnetococcus | **0** | **1** | **0** | **0** |
| MSBL2 | **0** | **2** | **9** | **0** |
| MSBL4 | **0** | **5** | **3** | **0** |
| MSBL5 | **0** | **0** | **1** | **2** |
| MSBL6 | **0** | **2** | **19** | **0** |
| MSBL8 | **0** | **0** | **0** | **1** |
| MSBL12 | **0** | **1** | **22** | **0** |
| Nitrospirae | **0** | **6** | **0** | **0** |
| OD1 | **0** | **1** | **0** | **0** |
| OP11 | **0** | **1** | **0** | **0** |
| Planctomycetes | **0** | **5** | **4** | **0** |
| Prochlorococcus | **1** | **0** | **0** | **0** |
| SAR406 | **1** | **6** | **9** | **0** |
| SB1 | **0** | **0** | **1** | **3** |
| Verrucomicrobia | **0** | **5** | **2** | **0** |
| WS3 | **0** | **0** | **15** | **0** |
| **TOT** | **112** | **78** | **102** | **110** |
| **Archaea** | **TZ** | **UIF** | **LIF** | **BB** |
| Archaeoglobi | **0** | **0** | **0** | **1** |
| HC 2 | **0** | **0** | **4** | **10** |
| MBG A | **2** | **0** | **0** | **0** |
| Methanosarcinales | **0** | **1** | **40** | **0** |
| MG I | **124** | **49** | **21** | **0** |
| MG II | **1** | **1** | **0** | **0** |
| MSBL1 | **0** | **0** | **6** | **91** |
| SA1 | **0** | **0** | **0** | **2** |
| SA2 | **0** | **0** | **0** | **1** |
| TMEG | **0** | **0** | **1** | **0** |
| VC2.1 Arc6 | **0** | **0** | **4** | **0** |
| **TOT** | **127** | **51** | **76** | **105** |
| **TOT (Bacteria+Archaea)** | **239** | **129** | **178** | **215** |

**Supplementary Table S5. Diversity indices calculated for the eight clone libraries of the Lake *Medee*.** Abbreviations used: TZ, transition zone; UIF, upper interface; LIF, lower interface; BB, Body brine. B: Bacteria; A Archaea, For statistical analyses and during clustering, the clones of each library were separately considered to define phylotypes at 98% and 97% of sequence identity.

| **Clone library** | **Taxa** | **Individuals** | **Dominance** | **Simpson (*D*)** | **Shannon (*H*)** | **Equitability (*E*)** | **Coverage (*c*)** | **singletons** | **doubletons** | **chao1** | **chao2** |
| --- | --- | --- | --- | --- | --- | --- | --- | --- | --- | --- | --- |
| B TZ (98%) | 9 | 112 | 0.739 | 0.260 | 0.633 | 0.288 | 0.936 | 7 | 0 | -- | 30.000 |
| B UIF (98%) | 48 | 78 | 0.031 | 0.968 | 3.704 | 0.956 | 0.623 | 29 | 14 | 78.036 | 75.067 |
| B LIF (98%) | 41 | 102 | 0.062 | 0.938 | 3.229 | 0.871 | 0.747 | 26 | 6 | 97.333 | 87.429 |
| B BB (98%) | 20 | 110 | 0.137 | 0.862 | 2.406 | 0.803 | 0.954 | 5 | 5 | 22.500 | 21.667 |
| B TZ (97%) | 9 | 112 | 0.739 | 0.260 | 0.633 | 0.288 | 0.936 | 7 | 0 | -- | 30.000 |
| B UIF (97%) | 47 | 78 | 0.032 | 0.967 | 3.662 | 0.951 | 0.610 | 30 | 10 | 92.000 | 86.545 |
| B LIF (97%) | 40 | 102 | 0.061 | 0.937 | 3.215 | 0.869 | 0.737 | 25 | 6 | 92.083 | 82.857 |
| B BB (97%) | 18 | 110 | 0.158 | 0.841 | 2.241 | 0.775 | 0.944 | 6 | 2 | 27.000 | 23.000 |
| A TZ (98%) | 17 | 127 | 0.155 | 0.844 | 2.184 | 0.770 | 0.960 | 5 | 4 | 20.125 | 19 |
| A UIF (98%) | 10 | 51 | 0.167 | 0.832 | 1.982 | 0.860 | 0.943 | 3 | 0 | -- | 13 |
| A LIF (98%) | 8 | 76 | 0.269 | 0.730 | 1.591 | 0.765 | 0.986 | 1 | 1 | 8.5 | 8 |
| A BB (98%) | 29 | 105 | 0.0645 | 0.935 | 3.032 | 0.900 | 0.922 | 8 | 8 | 33 | 32.111 |
| A TZ (97%) | 12 | 127 | 0.183 | 0.816 | 1.965 | 0.790 | 0.992 | 1 | 3 | 12.166 | 12 |
| A UIF (97%) | 9 | 51 | 0.186 | 0.813 | 1.867 | 0.849 | 0.943 | 3 | 0 | -- | 12 |
| A LIF (97%) | 8 | 76 | 0.27 | 0.73 | 1.58 | 0.759 | 0.973 | 2 | 0 | -- | 9 |
| A BB (97%) | 28 | 105 | 0.0653 | 0.937 | 3.007 | 0.902 | 0.932 | 7 | 8 | 31.062 | 30.333 |

**Supplementary Table S6**. **List of PCR primers used through the study.**

| Gene | Primer | Sequence (5’-3’) | Annealing site | Fragment (bp) | Reference |
| --- | --- | --- | --- | --- | --- |
| *16S rRNA bacteria* | 530F | GCC AGC AGC CGC GGT AAT AC | 530 -546 ^a^ | 982 | S1 |
|  | 1492R | TAC GYT ACC TTG TTA CGA CTT | 1492-1512 ^a^ |  |  |
| *16S rRNA archaea* | A20F | TTC CGG TTG ATC CYG CCR G | 20-38 ^b^ | 938 | S2, S3 |
|  | 958R | YCC GGC GTT GAM TCC AAT T | 940-958 ^b^ |  |  |
| *cbbL* | cbbl595F | GAC TTC ACC AAA GAC GAC GA | 595-614 ^c^ | 790 | S4 |
|  | cbbl1387R | TCG AAC TTG ATT TCT TTC CA | 1366-1385 ^c^ |  |  |
| *mcr* | MCRA F52 | GCT GCA TAC ACC AAC AAY AT | 52-71 ^d^ | 368 | S5 |
|  | MCR R420 | CCA CAC TGG TCY TGC ARG TC | 400-420 ^d^ |  |  |
| *dsrAB* | 1FI | CAG GAY GAR CTK CAC CG | 500 ^e^ | 1000 | S6 |
|  | 1RI | CCC TGG GTR TGR AYR AT | 1500 ^e^ |  |  |
| *apr* | AprA-1-FW | TGG CAG ATC ATG ATY MAY GG | 1236-1256 ^f^ | 395 | S7 |
|  | AprA-5-RV | GCG CCA ACY GGR CCR TA | 1615-1631 ^f^ |  |  |
| *acl* | Acl F892 | TGG ACM ATG GTD GCY GGK GGT | 892-912 ^g^ | 312 | S8 |
|  | Acl R1204 | ATA GTT KGG SCC ACC TCT TC | 1185-1204 ^g^ |  |  |

Corresponding nucleotide positions of the:

^a^*Escherichia coli,* ^b^*N. maritimus* SCM1 (NC_010085), ^c^*Anabaena* sp. strain 7120, ^d^*Methanohalophilus sp.,* ^e^*Desulfatibacillum* *alcenivorans* (AK01); ^f^*Desulfovibrio vulgaris* subsp. *vulgaris* strain Hildenborough (Z69372); ^g^*Alvinella pompejana* (7G3 fosmid clone).

**Supplementary Table S7.** Details of CARD-FISH probes and conditions used.

| **Probes** | **Target organism** | **Sequences**  **(5'-3')** | **FA^a^**  **(%)** | **HT^b^**  **(°C)** | **WT^c^**  **(°C)** | **Reference** |
| --- | --- | --- | --- | --- | --- | --- |
|  |  |  |  |  |  |  |
| Eub338 I | Bacteria | GCT GCC TCC CGT AGG AGT | 35 | 46 | 48 | S9 |
| Eub338 II | Bacteria | GCA GCC ACC CGT AGG TGT | 35 | 46 | 48 | S10 |
| Eub338III | Bacteria | GCT GCC ACC CGT AGG TGT | 35 | 46 | 48 | S10 |
| Arch915 | Archaea | GTG CTC CCC CGC CAA TTC CT | 35 | 46 | 48 | S11 |
| Eps914 | EPB | GGT CCC CGT CTA TTC CTT | 55 | 35 | 37 | this study ^d^ |
| Delta-DHAL | DPB | TCT AGC GCC CAA TGT TTA CG | 20 | 37 | 38 | this study ^d^ |
| Cren537 | MGI | TGA CCA CTT GAG GTG CTG | 0 | 46 | 48 | S12 |
| Eury806 | MGII | CAC AGC GTT TAC ACC TAG | 20 | 37 | 38 | S12 |
| Halo1192 | MSBL1/  Haloarchaea | GTA GCC CGC GTG TTG CCC GG | 20 | 46 | 48 | this study ^d^ |
| MSBL411 | MSBL1 | AGC CGA CGR TYG TTA GAC CA | 20 | 42 | 43 | this study ^d^ |
| KB1Eber | KB1 | GCA AAG CTT GAG GTC GTT CCC | 20 | 46 | 48 | this study ^d^ |
| NonEub338 | EUB Control | ACT CCT ACG GGA GGC AGC | 35 | 46 | 48 | S13 |
|  |  |  |  |  |  |  |

^a^ FA, Formamide concentration in hybridization buffer

^b^ HT, Hybridization temperature

^c^ WT, Washing temperature

^d^ The hybridization conditions for the newly designed probes were optimized (formamide concentrations tested: 0, 15, 20, 25, 30, 35, 50 and 60%) and the optimal FA concentration is present. Unspecific binding of the newly designed probes was checked using SW, TZ and UIF filters lacking these groups of prokaryotes.

**Supplementary Figures.**

**Figure S1. CTD depth profiling of temperature, salinity and oxygen concentration across the adjacent seawater, ‘transition zone’, interface and brines of the Lake *Medee* analysed during cruises Middle&Mamba (September, 2009) and MicroDeep (September, 2012) (a and b, respectively).**

While entering in hypersaline anoxic layers, oxygen and salinity values surpassed the calibration range of the CTD sensors. Their drastic change was used through the present study for positioning of TZ and interface.

**
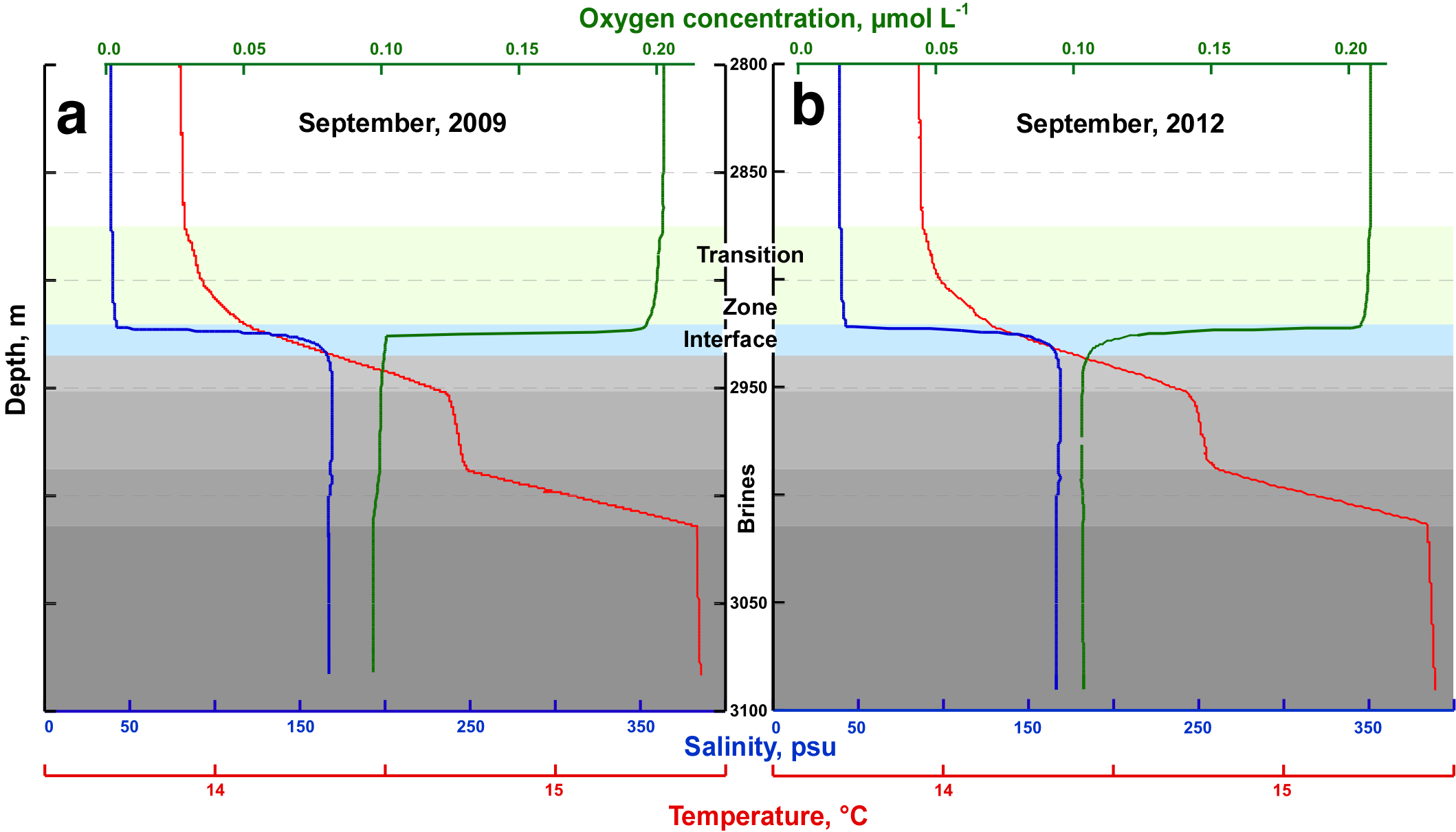
**

**Figure S2a. Phylogenetic affiliation of *Medee* eubacterial clones: *Proteobacteria* and MSBL6.**

See Text S4 for details on phylogenetic analyses. Neighbor-joining analysis using 1000 bootstrap replicates was used to infer tree topology. The scale bar represents 10% of sequence divergence. Bootstrap values are indicated at branch points as open (>50%) and closed (>75) circles. Sequences obtained in this study are indicated in bold.

**Figure S2b. Phylogenetic affiliation of *Medee* eubacterial clones: from MSBL6 to SAR406**.

**Fig. S2c. Phylogenetic affiliation of *Medee* eubacterial clones: from JS1 to OP1.**

**Fig. S3. Phylogenetic affiliation of *Medee* archaeal clones.**

See Text S5 for details on phylogenetic analyses. Neighbor-joining analysis using 1000 bootstrap replicates was used to infer tree topology. The scale bar represents 10% of sequence divergence. Bootstrap values are indicated at branch points as open (>50%) and closed (>75%) circles. Sequences obtained in this study are indicated in bold.

**Fig. S4. Overview on diversity (a), stratification and relative abundance (b) of cbbL clones recovered from the different compartments of *Medee* Lake.**

The tree was constructed by Neighbour-Joining method and Gonnet distance matrix with Poisson correction using the MacVector 11.1.2 software. Bootstrap values >70% are shown as filled circles and were calculated over 1000 random repetitions. Sequences obtained in this study and in other Mediterranean DHALs are indicated in bold. The scale bar represents 10% sequence divergence.

**
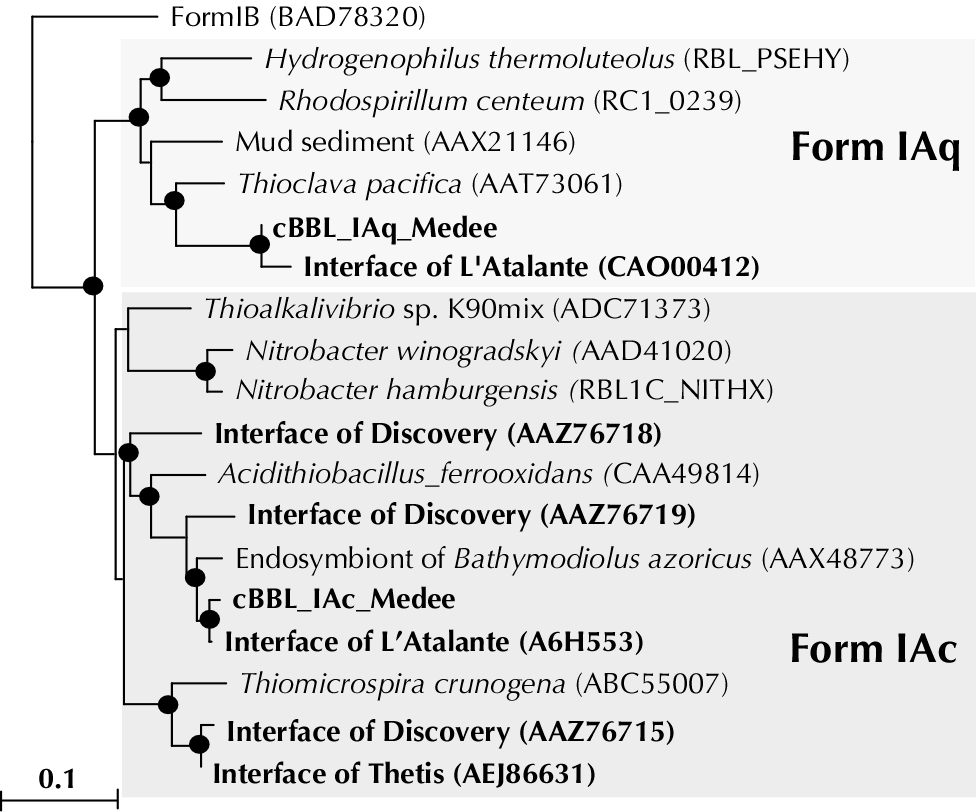
**

**Fig. S5. Diversity and stratification of sulfur-oxidizing bacteria (SOB) and sulfur-reducing prokaryotes (SRP) as revealed by phylogenetic affiliation of *aprA* transcripts recovered from different layers of the *Medee* Lake.**

(a.) The tree was constructed by neighbour-joining method and Jukes–Cantor distance matrix using the MEGA software. DHAL sequences are indicated in bold. Sequences obtained from transition zone, upper interface, middle interface and body brine are indicated in orange, green, blue and red, respectively. ThermoDB stands for *Thermodesulfurobacteria*-related sequences. The scale bar represents 9% sequence divergence. (b.) Abbreviations of *aprA* groups correspond to those in the tree: APB, *Alphaproteobacteria*; D-R, deep rooting cluster with unclear affiliation; DPB, *Deltaproteobacteria;* F, *Firmicutes*; GPB, *Gammaproteobacteria*; SOB, sulfur-oxidizing bacteria; SRP, sulfate-reducing prokarytoes; TSB, *Thermodesulfurobacteria*. Layer-specific groups are highlighted in gold.


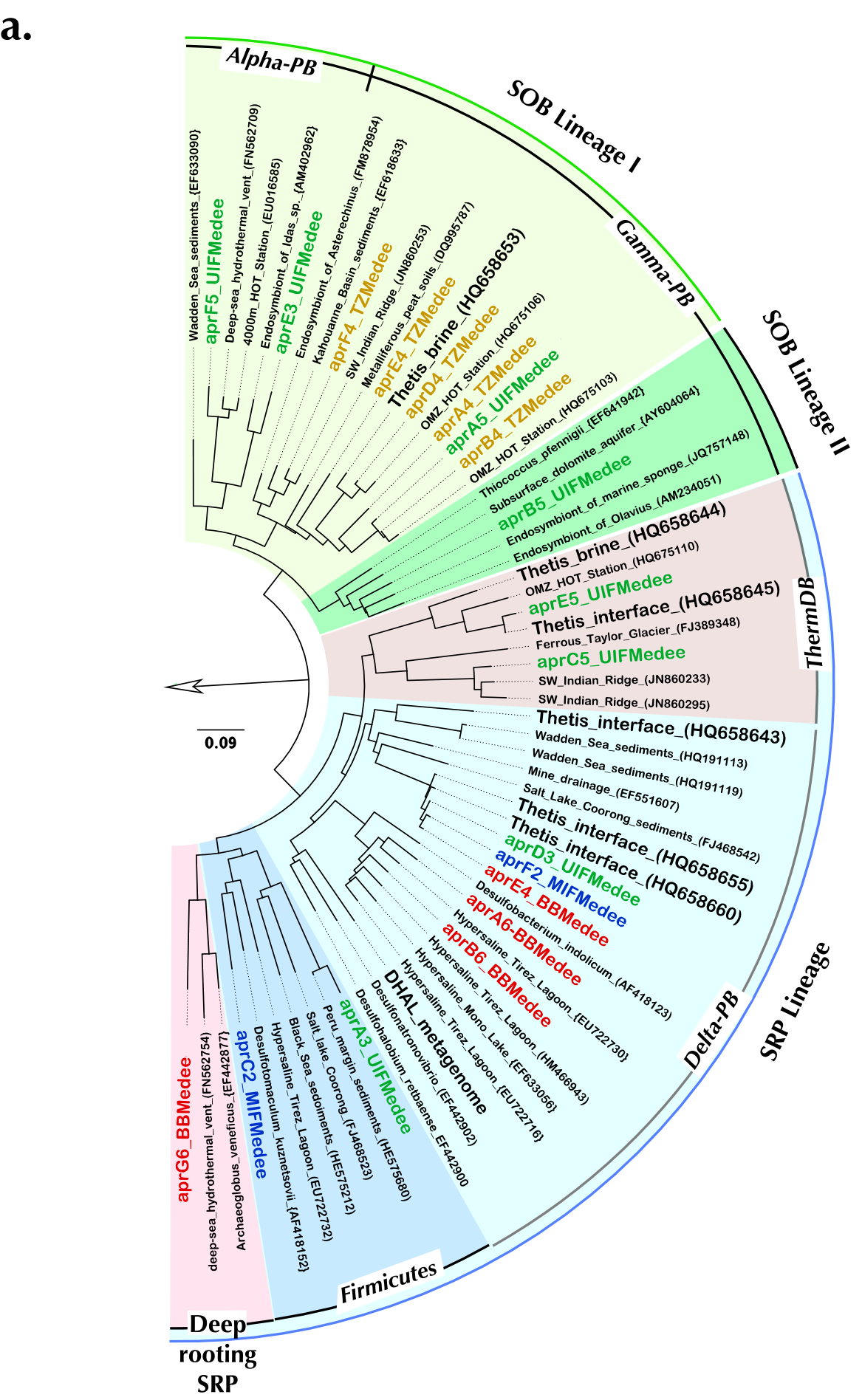


**
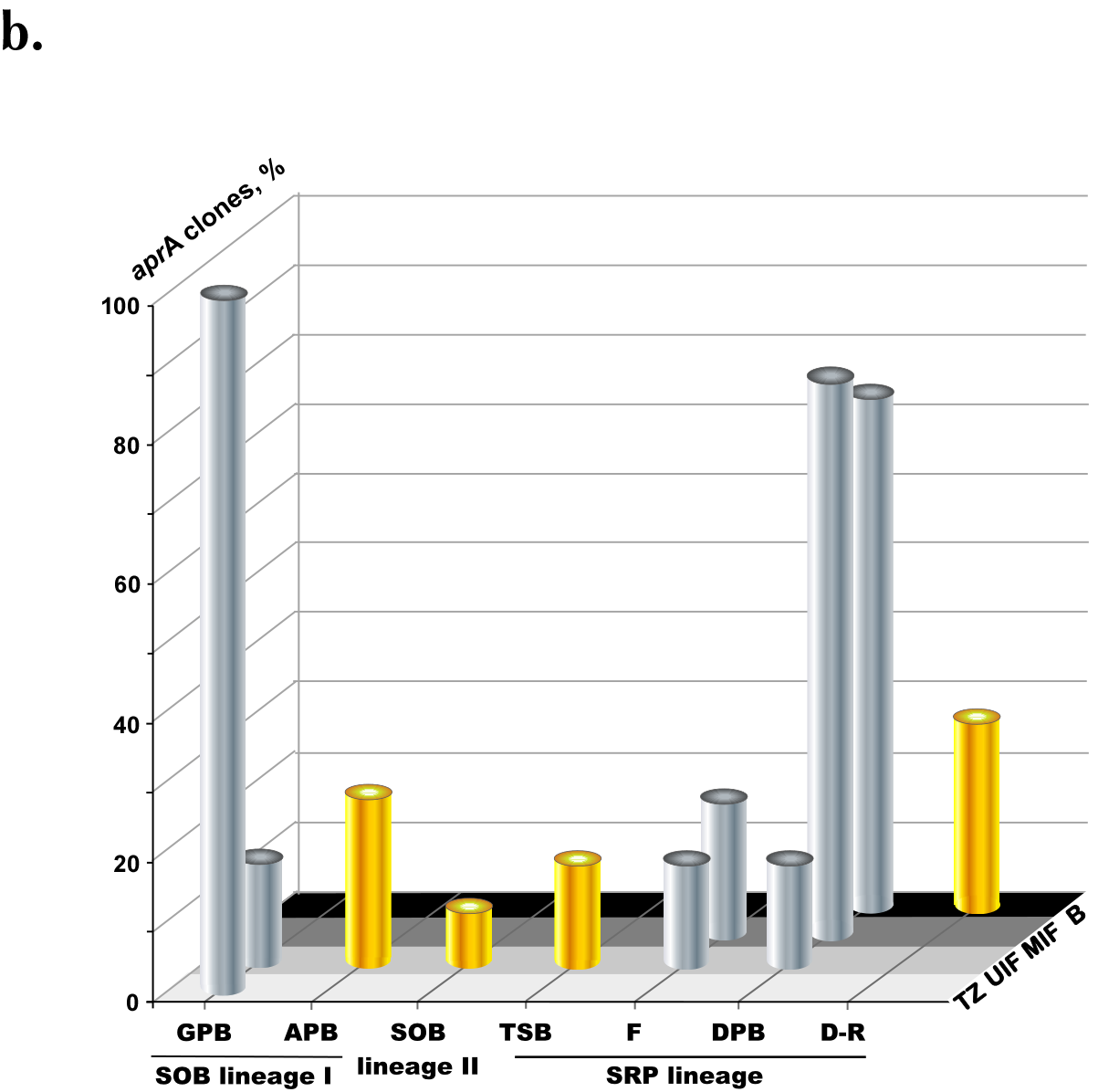
**

**Fig. S6. Direct microscopic observations of prokaryotic communities of CARD-FISH- and DAPI-stained cells collected in Lake *Medee* brine.**

Polymorphic single cells and chains of rod-shaped cells positive for the KB1 probe (a and b), vibrio-shaped Delta-DHAL-positive cells (c) and MSBL1-positive cells (d) were observed in Lake *Medee* brine at the depth of 3,010m bsl. Right column represents the overlay of DAPI-stained (central column) and Cy3-stained cells images (left column). Red scale bars denote: 5µm.


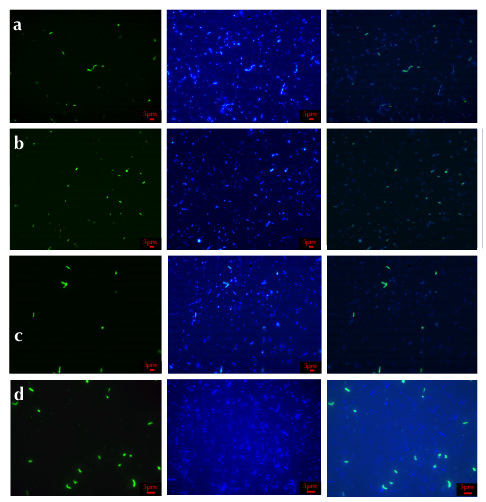


**Fig. S7. Phylogenetic tree based on *mcrA* cDNA sequences recovered from the Lake *Medee*.** The trees were constructed by neighbour-joining method and Jukes–Cantor distance matrix using the MEGA software. Bootstrap values >70 are shown as filled circles and were calculated over 1000 random repetitions. Sequences obtained from other Mediterranean DHALs and in this study are indicated in bold. The scale bar represents 10% sequence divergence.


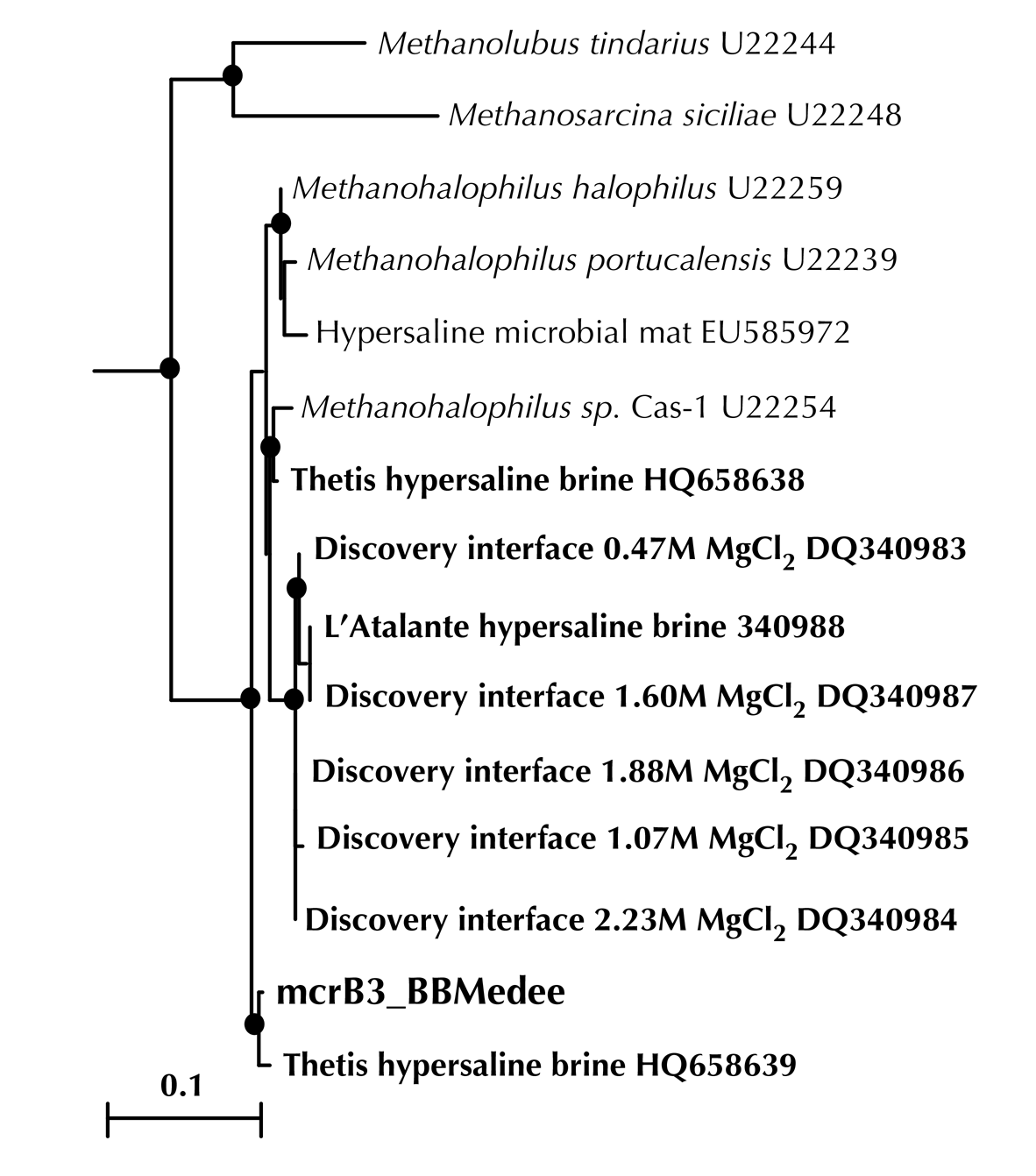


**Fig. S8. Phylogenetic analyses of GB- and TMA-enriched microbial communities of *Medee* brine.**

16S crDNA sequences recovered from *Medee* brine and GB- and TMA-enrichments are evidenced respectively by blue and red and violet colors, whereas sequences recovered from other DHALs are shown in black. Bootstrap values >60% and >75%are shown as open and filled circles and were calculated over 1000 random repetitions. Scale bar indicates distance in length (10% sequence divergence).


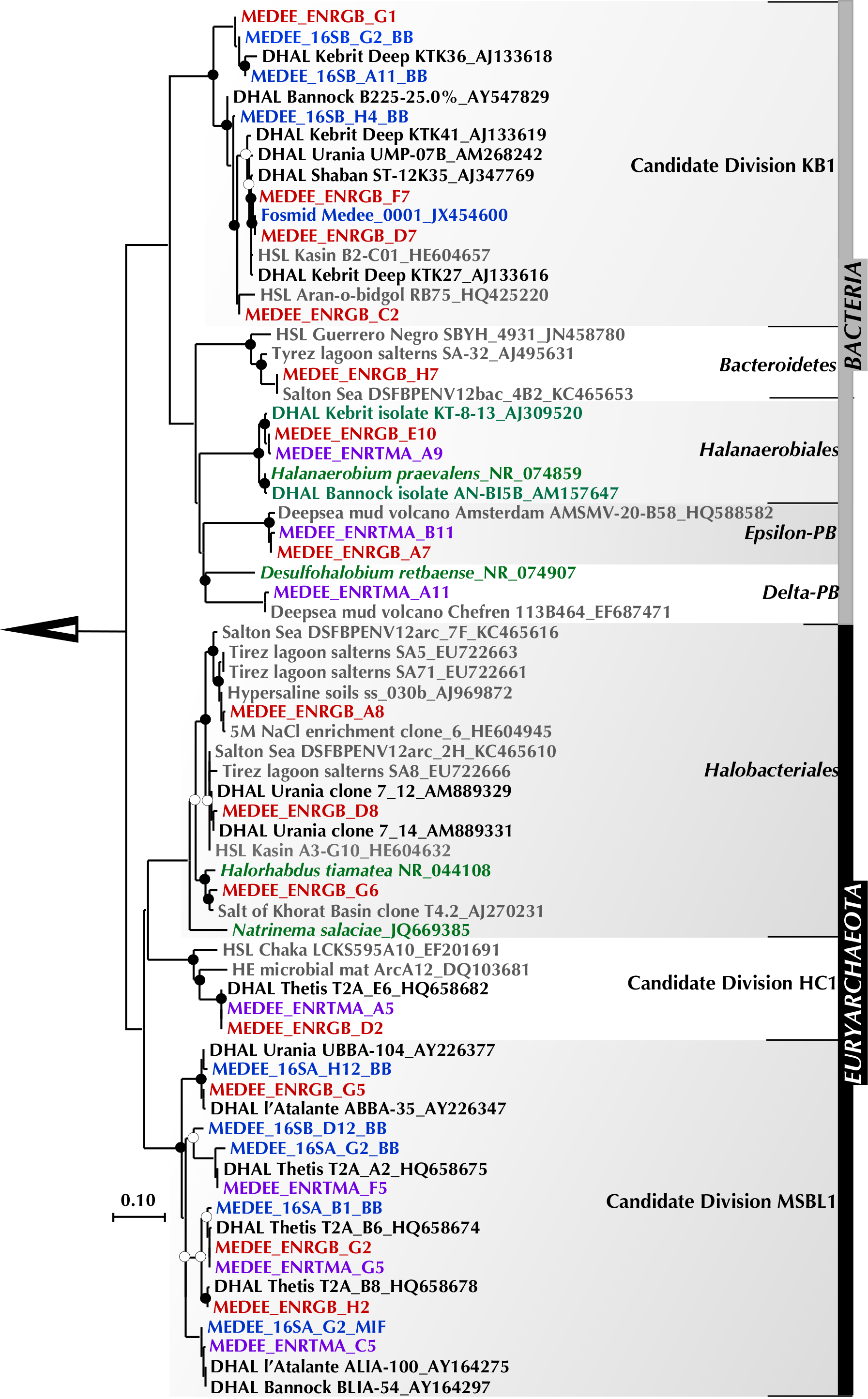


**Supplementary Texts and Supplementary References.**

**Text S1. Extended Protocol for uptake of [methyl-^14^C]-glycine betaine (GB).**

For anaerobic uptake of GB in the brine (salinity, 320) and lower interface (salinity, 265) 100 mL of each sample were used in triplicate and one formaldehyde-fixed blank control. The experiment was performed into serum vials (120 mL) carefully flushed with argon to remove any oxygen. An anoxic [methyl-^14^C]-glycine betaine stock solution (51.9 mCi mmol^-1^; 100 Ci mL^-1^; Moravek Biochemicals & Radiochemicals, Brea, CA) was diluted forty times in artificial brine (280 g L^-1^ of NaCl) and 1 mL of this solution corresponding to 2.5 µCi or 482 nmol L^-1^ of GB was added to the serum vials using a gas-tight Hamilton syringe. The applied concentration of GB was comparable to those found in the upper brine of *Medee* Lake (Table 1). Samples were incubated for 30 days at *in situ* temperature (16 °C) in the dark. Metabolic inhibitors of methanogenesis (100 mmol L^-1^ bromoethanesulfonate) and sulphate reduction (20 mmol L^-1^ sodium molybdate) were diluted in artificial brine and added to 100 mL of each sample used in triplicate. Incubation was stopped by the addition of formaldehyde to a final concentration of 2% (v/v) and samples were filtered onto polycarbonate membrane filters (0.1 μm pore size, Millipore). Filters were washed three times with 10 mL of ultra-filtered (0.1 μm) artificial brine, mixed with scintillation cocktail (Ultima Gold™ MV, PerkinElmer), and counted in a liquid scintillation counter (Wallac WinSpectral 1414 Liquid Scintillation Counter, PerkinElmer Life Sciences) using the internal radioisotopes library and quenching correction. The values obtained in disintegrations per minute were normalized against the values of the abiotic control and amount of incorporated [methyl-^14^C]-GB was calculated according the ratio 1nCi = 2,220 dpm.

**Text S2. Experimental Protocol for dark CO_2_ fixation and prokaryotic heterotrophic production (PHP).**

For aerobic-microaerophilic incubations (deep WC and TZ), 40 mL for each sample in triplicate and one formaldehyde-fixed blank control were used. The determination of CO_2_ dark fixation rates for the micro-aerobic and anoxic samples (upper- and middle-interfaces and body brine) was performed into serum vials (120 mL) carefully flushed with argon to remove any oxygen. An anoxic sodium [^14^C]-bicarbonate stock solution (250 Ci mL^-1^) was added to the serum vials using a gas-tight Hamilton syringe, thereby only insignificantly altering the total inorganic carbon concentration of about 2 mmol L^-1^. Samples were incubated for 10 days at *in situ* temperature in the dark. Incubation was stopped by the addition of formaldehyde to a final concentration of 2% (v/v) and samples were filtered onto polycarbonate membrane filters (0.1 μm pore size, Millipore). Filters were washed with 10 ml of ultra-filtered (0.1 μm) seawater, exposed to HCl fumes (12 hours), mixed with scintillation cocktail (Ultima Gold™ MV, PerkinElmer), and counted in a liquid scintillation counter (Wallac WinSpectral 1414 Liquid Scintillation Counter, PerkinElmer Life Sciences) using the internal radioisotopes library and quenching correction. The values obtained in disintegrations per minute were normalized against the values of the abiotic control and corrected for the natural DIC. Triplicate subsamples and duplicate blanks were incubated with 20 nmol of leucine (5 nmol L-[4,5- ^3^H]-leucine, SA= 165.2 Ci mmol^-1^ + 15 nmol L-leucine), in the dark, during 150 min at "*in situ*" ± 1.5 °C temperatures. Heterotrophic prokaryotic carbon biomass production was calculated according to Kirchman (1993) using in situ determinations of leucine isotopic dilution (ID) calculated according to Pollard and Moriarty (1984). The incorporated radioactivity was measured as DPM counts with a liquid scintillation counter (Wallac WinSpectral 1414 Liquid Scintillation Counter, PerkinElmer Life Sciences) using the internal radioisotopes library and quenching correction.

Kirchman, D.L., Keil, R.G., Simon, M. & Welschmeyer N. A. Biomass and production of heterotrophic bacterioplankton in the oceanic subarctic Pacific. *Deep Sea Res. Part I Oceanogr. Res. Pap.* **40**, 967–988 (1993).

Pollard, P. C. & Moriarty, D. J. W. Validity of isotope dilution of tritiated thymidine during incorporation into DNA as an estimate of bacterial growth rates. *Appl. Environ. Microbiol*. **48**, 1076-1083 (1984).

**Text S3. Extended experimental protocol for *in situ* determination of GB and methylated amines.**

Amino acids fingerprinting and concentrations of GB and methylated amines were determined by using LC-ESI-QTOF. Briefly the metabolic profile was achieved by a Liquid Chromatography system consisted of a degasser, one binary pump and an auto-sampler (1290 infinity, Agilent). 0.5 µL was applied to a reversed-phase column (Zorbax Extend C_18_ 50 x 2.1 mm, 3 μm; Agilent), which it was kept at 60 ºC during the analysis. The system was operated in positive and negative ion mode at a flow rate 0.6 mL/min with solvent A composed of water with 0.1% formic acid and solvent B composed of acetonitrile with 0.1% formic acid. The gradient was: 5 % B (0-1 min), 5 to 80 % B (1-7 min), 80 to 100 % B (7-11.5 min) and 100 to 5 % B (11.5-12 min), keeping the re-equilibration at 5 % B for 3 min (15 min of total analysis time). Data were collected in positive and negative ESI mode in separate runs on a QTOF (Agilent 6550 iFunnel). For positive mode it operated in full scan mode from 50 to 1000 m/z. The capillary voltage was 3000 V with a scan rate of 1.0 spectra per second. The gas temperature was 250 ^o^C, the drying gas flow 12 L/min and the nebulizer 52 psi. For negative mode it operated in full scan mode from 50 to 1100 m/z. The capillary voltage was 3000 V with a scan rate of 1.0 spectra per second. The gas temperature was 250 ^o^C, the drying gas flow 12 L/min and the nebulizer 52 psi. During the positive analysis, two reference masses were used: 121.0509 (C_5_H_4_N_4_) and 922.0098 (C_18_H_18_O_6_N_3_P_3_F_24_) as well as in negative: 112.9855 (C_2_O_2_F_3_) and 1033.9881 (C_18_H_18_O_6_N_3_P_3_F_24_). They were continuously infused to the system to allow constant mass correction. Samples were analyzed in randomized run, during this time they were kept in the LC auto-sampler at 4^o^C. The resulting data files were cleaned of background noise and unrelated ions by the Molecular Feature Extraction tool in the Mass Hunter Qualitative Analysis software (B.05.00, Agilent). The total TIC (Total Ion Chromatogram) was examined for GB and TMA MS signatures. GB concentration was determined using GB standard (Sigma-Aldrich, Taufkirchen, Germany) with a concentration ranging from 1 to 10 µmol L^-1^.

**Text S4. Experimental Protocol for phylogenetic reconstruction.**

Sequences were checked for possible chimeric origin using Bellerophon software (S14). For the 16S rRNA gene sequences, initial alignment of amplified sequences and close relatives identified with BLAST (S15) were performed using the SILVA alignment tool (S16) and manually inserted in ARB (S17). After alignment, the neighbour-joining algorithm and Jukes-Cantor distance matrix of ARB program package was used to generate the phylogenetic trees based on distance analysis for 16S rRNA. 1000 bootstrap resamplings were performed to estimate the reproducibility of the partitions in the tree. For statistical analyses clones of each library were separately considered to define phylotypes at 97% of identity, using DNADIST of the Phylip package (<http://evolution.genetics.washington.edu/phylip.html>).

**Text S5.** Experimental Protocol for rarefaction analysis, diversity index and coverage values of analyzed clone libraries.

For statistical analyses clones of each library were separately considered to define phylotypes at 98% and 97% of sequence identity. The presence of library-specific phylotypes in other libraries were further analysed by total alignment. Clustering of sequences was performed using Dotur program (S19), diversity index: Rarefaction analysis, Dominance, Coverage (c), Shannon (H), Equitability (E), Simpson (D)] chao1-2 index for each clone library were performed using Past (Paleontological Statistics 2.16) (S20). Coverage values were calculated to determine how efficient our clone libraries described the complexity of a theoretical community such as original bacterial community. The coverage value is given as C = 1 − (n_1_ / N) where n_1_ is the number of clones which occurred only once in the library (singleton) (S21). Number of missing species was calculated using Chao2 index: S = D + f_1_ * (f_1_-1) / 2 * (f_2_+1) were S, total number of species in a community; D, number of distinct species discovered in the sample, f_k_= number of species that are represented exactly k times in the sample (S22).

Dominance, Shannon, Equitability, Simpson indexes, as well as the coverage, Chao1 and Chao2 values calculated for each eubacterial library. The rarefaction analysis was carried out to determine whether a sufficient number of clones from each of libraries was sequenced. Applying the cut-off value of <97% of sequence identity, the generated curves obtained from bacterial clone libraries did not demonstrate the saturation for each library. The bacterial biodiversity is strongly affected by the stratification of the basin: the transition zone present the lowest value of biodiversity (Shannon 0.633, Equitability 0.288) together with high values of coverage (0.936) and the dominance of a few bacterial groups (Dominance 0.739), mainly *Alteromonadaceae*. The interface shows the maximum of biodiversity, especially UIF (Shannon 3.62 and Equitability 0.951), and the highest numbers of missing species calculated according to Chao (1984) (>80.00). Archaeal biodiversity presents a pattern significantly different than those of the *Bacteria*. The biodiversity values remain moderately constant between the transition zones and interface (dominance ~0.186, Simpson ~0.813, Shannon 1.867) whilst they tend to increase significantly in the brine body (Simpson 0.937, Shannon 3.007). Higher value of coverage (>0.9) and rarefraction analysis performed with archaeal libraries revealed that their diversity was sufficiently covered along the different depths analyzed.

**Supplementary References:**

S1. Lane, D. J. (Eds) 16S/23S rRNA Sequencing. In *Nucleic Acid Techniques in Bacterial Systematics,* Stackerbrandt E. and Goodfellow, M. Chichester, UK: John Wiley&Sons Ltd, pp. 115-175 (1991).

S2. DeLong, E. F. Archaea in coastal marine environments. *Proc. Natl. Acad. Sci. USA* **89**, 5685- 5689 (1992).

S3. Stackebrandt, E., & Goodfellow, M. Nucleic acid techniques in bacterial systematics. Wiley, Chichester, England (1991).

S4. Elsaied, H., & Naganuma, T. Phylogenetic diversity of ribulose-1,5-bisphosphate carboxylase/oxygenase large subunit genes from deep-sea microorganisms. *Appl. Environ.* *Microbiol.* **67**, 1751-1765 (2001).

S5. Hallsworth, J. E., *et al. Limits of life in MgCl2-containing environments: chaotropicity defines the window*. *Environ. Microbiol.* **9**, 801-813 (2007).

S6. Dhillon, A., *et al.* Molecular characterization of sulfate-reducing bacteria in the Guaymas Basin. *Appl. Environ. Microbiol.* **69**, 2765-2772 (2003).

S7. Meyer, B. & Kuever, J. Molecular analysis of the distribution and phylogeny of dissimilatory adenosine-5’- phosphosulfate reductase-encoding genes (aprBA) among sulfur oxidizing prokaryotes. *Microbiology* **153**, 3478–3498 (2007).

S8. Campbell, B. J., Stein, J. L., & Cary S. C. Evidence of chemolithoautotropy in the bacterial community associated with Alvinella pompejana, a hydrothermal vent polychaete. *Appl. Environ. Microbiol.* **69,** 5070–5078 (2003).

S9. Amann, R.I. *et al.* Combination of 16S rRNAtargeted oligonucleotide probes with flow cytometry for analyzing mixed microbial populations. *Appl. Environ. Microb.* **56**, 1919-1925 (1990).

S10. Daims, H. *et al.* The domain specific probe EUB338 is insufficient for the detection of all Bacteria: Development and evaluation of a more comprehensive probe set. *Syst Appl Microbiol* **22**, 434-444 (1999).

S11. Stahl, D. A. & Amann, R. (Eds) Development and application of nucleic acid probes. In *Nucleic Acid Techniques in Bacterial Systematics*. Stackebrandt E. and Goodfellow M. Chichester, UK: John Wiley&Sons Ltd, pp. 205-248 (1991).

S12. Teira, E. *et al.* Combining catalyzed reporter deposition-fluorescence in situ hybridization and microautoradiography to detect substrate utilization by Bacteria and Archaea in the deep ocean. *Appl. Environ. Microb.* **70**, 4411–4414 (2004).

S13. Wallner, G., Amann, R., & Beisker, W. Optimizing fluorescent in situ hybridization with rRNA targeted oligonucleotide probes for flow cytometric identification of microorganisms. *Cytometry* **14**, 136-143 (1993).

S14. Huber, T., Faulkner, G. & Hugenholtz, P. Bellerophon: a program to detect chimeric sequences in multiple sequence alignments. *Bioinformatics* **20**, 2317–2319 (2004).

S15. Altschul, S.F. *et al.* Gapped BLAST and PSI-BLAST: a new generation of protein database search programs. *Nucleic Acids Res* **25**, 3389–3402 (1997).

S16. Pruesse, E. *et al.* SILVA: a comprehensive online resource for quality checked and aligned ribosomal RNA sequence data compatible with ARB. *Nucleic Acids Res* **35**, 7188–7196 (2007).

S17. Ludwig, W. *et al.* ARB: a software environment for sequence data. *Nucleic Acids Res* **32**, 1363–1371 (2004).

S18. Giovannoni, S. Evolutionary biology: oceans of bacteria. *Nature* **430**, 515–516 (2004).

S19. Schloss, P.D., Handelsman, J. Introducing DOTUR. a computer program for defining operational taxonomic units and estimating species richness. *Appl Environ Microbiol* **71**, 1501–1506 (2005).

S20. Hammer, Ø., Harper, D.A.T., & Ryan, P.D. PAST: Paleontological statistics software package for education and data analysis. *Palaeontologia Electronica* **4**, 9p. (2001).

S21. Good, I.J. The population frequencies of species and the estimation of the population parameters. *Biometrika* **40**, 237–264 (1953).

S22. Shen, T.-J., Chao, A., and Lin, J.-F. Predicting the number of new species in further taxonomic sampling. *Ecology*, **84**, 798-804 (2003).
